# Paired mutation calling and spatial transcriptomics identify cellular neighbourhoods dictating the neoplastic outcome of colitis

**DOI:** 10.21203/rs.3.rs-6401505/v1

**Published:** 2025-05-08

**Authors:** E. B. Moutin, L. Chang, G. Giavara, S. Mehmed, M. Colombé, F. C. Lourenço, M.N. Skoufou-Papoutsaki, R. Kemp, P. Gascard, T. Tlsty, D.S. Tourigny, D.J. Winton

**Affiliations:** 1 CRUK Cambridge Institute, University of Cambridge; 2 School of Mathematics, University of Birmingham; 3 Department of Pathology, University of California San Francisco

## Abstract

In the progression from Inflammatory Bowel Disease to associated cancer, the clonal mutational landscape shifts from selection of mutations in inflammatory genes to selection of cancer-driver mutations^1–4^. How prevalence and expansion of either type of mutated clones could be impacted by the cellular environment in which they arise, and how this affects the neoplastic outcome of colitis is unknown. Here, we combine *in vivo* lineage tracing, *in-silico* modelling, mutational profiling and spatial transcriptomics in a mouse model of colitis-associated tumorigenesis to capture clone fates associated with chronic inflammation. We identify epithelial- and immune-enriched neighbourhoods and propose a model in which establishment of a reparative tissue environment facilitates tumours initiation by promoting the selection and expansion of pro-oncogenic clones, reducing the span of inflammation-resistant neighbourhoods containing non-oncogenic clones.

## INTRODUCTION

Inflammatory Bowel Disease (IBD) affects seven million people worldwide and is associated with many co-morbidities, including the development of colitis-associated-cancer (CAC)^5^. During the progression of IBD, mutations affecting genes implicated in mounting an inflammatory response – including those of the IL17 and NF-KB pathways – are under positive selection in the colonic epithelium^1–3^. Clones bearing such mutations exhibit an increased capacity to survive and expand in colonic tissue, which has been linked with resistance to inflammatory insult. In contrast, tumours arising in the context of IBD show reduced prevalence of inflammation-resistant mutations, suggesting a protective role against cancer that could explain why most patients are only diagnosed with colorectal neoplasia more than a decade after their initial IBD diagnosis^6,7^. How selection of inflammation-resistant clones is influenced by the environment in which they arise and how this affects the balance between IBD pathogenesis and neoplastic transformation has not yet been defined, in part due to a lack of model systems.

Using lineage tracing to profile clone dynamics in the well-characterised *Muc2^KO^* model of colitis^8–10^, we observe discrete regions in which clonal expansion is favoured. *In-silico* modelling of clone dynamics and inference of effective fission rate identifies tissue repair as the process driving neutral clonal expansion through increased crypt fission. The impact of expansion of mutated clones in this context was studied using targeted amplicon sequencing after chemical mutagen treatment of *Muc2^KO^* mice. Importantly, clones bearing mutations found in human IBD and associated cancer were captured. Paired spatial transcriptomics defined the function of such mutated clones in a chronically inflamed context and captured the information necessary to define cellular neighbourhoods within which these clones preferably expand, dictating the neoplastic outcome.

## RESULTS

### Lineage tracing captures increased clonal expansion in colitis

**Figure 1:**
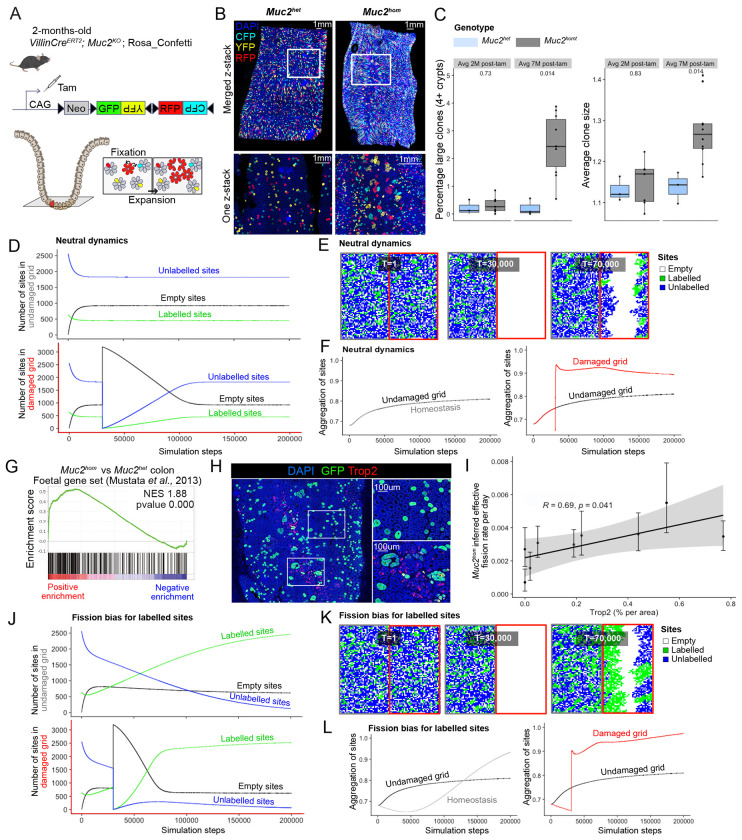
Impact of colitis-associated tissue repair on clonal fates. A) *In vivo* genetic lineage tracing using VillincreERT; Muc2KO; Rosa26flConfetti reporter mice. Tamoxifen treatment leads to Cre-mediated excision of the Neomycin cassette, resulting in heritable expression of either GFP, YFP, RFP or CFP in intestinal epithelial cells. Clones can achieve high intestinal epithelial burden by two processes: 1. fixation, relating to the capacity of stem cells to compete with others at the crypt base to achieve monoclonality, 2. expansion, relating to the capacity of a clone to fission into more crypts. B) 3D images (top) and one z-stack pictures (bottom) showing increased clone sizes in the *Muc2*^*hom*^ compared to *Muc2*^*het*^ mouse colon. C) Boxplot showing percentage of large clones (left) and average clone size (right) in animal groups for which average survival is either 2 months post-tamoxifen (Muc2^het^ n= 3, *Muc2*^*hom*^ n=7) or 7 months post-tamoxifen (Muc2^het^ n= 3, *Muc2*^*hom*^ n=10) (p values for two-sided Wilcoxon tests showed on plots). D) Line plots showing average number of labelled, unlabelled or empty sites per simulation step in each side of the lattice (top = no damage, bottom = damage at T=30,000) for 1,000 simulations in neutral dynamics conditions. E) Representative grid at simulation steps corresponding to beginning of simulation (T=1), introduction of damage (T=30,000) and ongoing repair (T=70,000). F) Line plot showing aggregation of sites in both sides of the grid as well as homeostatic aggregation per simulation step, averaged over 1,000 simulations (right = no damage, left = damage at T=30,000) . G) Gene Set Enrichment Analysis (GSEA) on bulk RNA sequencing material from *Muc2*^*hom*^ and *Muc2*^*het*^ mouse colon using a foetal gene set ^*46*^ (n=3 mice per condition). H) Immunofluorescence pictures showing confetti clones stained with GFP in a Trop2+ and Trop2− area of the *Muc2*^*hom*^ mouse colon. I) Dot plot showing linear correlation between percentage of area expressing Trop2 and inferred effective fission rate (n=3 *Muc2*^*hom*^ mice, 3 regions per mouse colon, coefficient and p value for Pearson correlation on plot). J) Line plot as in E, with fission bias in labelled sites (0.95 vs 0.5 unlabelled). K) Representative snapshots as in F for fission bias condition. L) Line plot showing aggregation of sites as in G for fission bias condition.

Depletion of the intestinal mucus barrier due to constitutive knockout of both alleles of the *Muc2* gene leads to chronic inflammation restricted to the mouse colon^11^. As previously described by us and others, this model recapitulates the pathology seen in patients suffering from Inflammatory Bowel Disease (IBD) ^9,10^, including the development of epithelial dysplasia and emergence of invasive foci with age (Supplementary Fig.1). To observe clonal fates in the colonic epithelium accompanying disease progression in this model, Cre-mediated activation of the Confetti lineage-tracing cassette was performed by tamoxifen treatment of mice containing inter-crossed *R26^Confetti^* and *VillinCre^ERt2^* alleles^12^ on a *Muc2^KO^* background (Fig. 1a). Large clones could be observed in discrete areas of the *Muc2*^hom^ mouse colon (Fig. 1b). Quantification of clone sizes at 7-months post tamoxifen injection revealed a significant increase in average clone size and number of large clones in the colonic epithelium of *Muc2*^hom^ mice compared to control *Muc2*^het^ mice, two phenotypes that were not apparent at 2 months post tamoxifen injection (Fig. 1c). This indicates a clear relationship between duration of inflammation and clonal expansion.

### Increased crypt fission drives reparative neutral clonal expansion

An *in-silico* model was developed to explore how discrete neighbourhoods containing clonal expansions might arise due to inflammatory injury. A square lattice was created to represent a colon segment cut longitudinally. The lattice was randomly populated with 80% unlabelled and 20% labelled sites (representing crypts) and evolved forward in time in a sequence of steps allowing the transition of sites between three possible states: empty, unlabelled or labelled. Transitions are constrained by the probability of clone fixation, crypt fission, and crypt fusion (Methods and Supplementary Note), all neutral in homeostasis ^13,14^.

After an initial transient state, the number of empty sites reached a plateau, considered here as homeostasis (Fig. 1d). To replicate the effects of acute damage, the entire right-half of the lattice was emptied shortly after reaching homeostasis, and sites from the left-hand grid allowed to repopulate the damaged grid (Fig. 1e). Of note, the damaged grid may be repopulated from both sides as it is a 2D representation of the cylindric shape of the colon. To capture the clonal expansion seen *in vivo*, we derived a metric quantifying aggregation of either labelled or unlabelled sites (Methods). Increased aggregation was seen in the right-hand grid post-damage, and persisted after homeostasis was reached (Fig. 1f), supportingthat repair after damage is achieved by neutral expansion of clones in proximity to injury.

To identify markers associating with epithelial repair, transcriptional profiles of *Muc2*^hom^ mouse colons were derived. Previous observations in the Dextran Sulphate Sodium (DSS) colitis model have identified a foetal gene signature characteristic of the repairing epithelium^15^. Enrichment for this signature was confirmed in the *Muc2*^hom^ model by gene set enrichment analysis (GSEA) (Fig. 1g). Immunodetection revealed presence of large clones in regions where the foetal marker Trop2 (*Tacstd2*) is expressed (Fig. 1h). Estimates of effective crypt fission rates showed a significant positive correlation to extent of Trop2 expression within the region profiled (Fig. 1i). Together, these results establish repairing areas of the mouse colonic epithelium as environments where clonal expansion through crypt fission is favoured. Notably, the restriction of this response to the damaged area reflects the localised pathology observed in Ulcerative Colitis patients.

### Tissue repair conditions favour fission-biased clones

Mutations in cancer-driving genes such as *Kras* have been shown to confer a large fission bias to mutant clones^16^. To study the potential impact of such biases on expansion in tissue repair conditions, *in-silico* simulations were adapted to give a fission advantage to labelled sites (fission probability = 0.95 for labelled sites versus 0.5 for unlabelled sites). This led to faster repair of the damaged right grid (T=80,000 simulation steps with fission advantage versus 115,315 without) (Fig. 1d, j), with fission-biased sites consistently outcompeting wild-type sites in both damaged and non-damaged grids (Fig. 1j, k). This was linked to a stable increase in aggregation of sites on both sides of the lattice, that was, importantly, more pronounced in the damaged side (Fig. 1l). This highlights how tissue repair conditions can accelerate and promote the accumulation and expansion of mutant clones in damaged tissue.

### Approach for combined profiling of mutations and cellular context in colitis-associated-cancer

To model a context in which mutated clones expand in chronically inflamed tissue, *Muc2*^hom^ mice were treated with the chemical mutagen N-ethyl-N-nitrosourea (ENU). This alkylating agent has been shown by us and others to trigger tumour initiation in primed contexts^17,18^. Upon ENU treatment, tumours arise in the colon of *Muc2*^hom^, but not *Muc2*^het^ mice (Supplementary Fig.1b), resulting in reduced survival in the former (Supplementary Fig.1c). These tumours arise as flat dysplastic lesions, phenocopying colitis-associated-cancer (Supplementary Fig. 1d). Notably, tumours express Notum and Reg4, that have been documented as markers of Wnt pathway activation and colitis-associated-cancer, respectively^19,20^. To determine how expansion of specific mutated clones is influenced by the environment in which they arise, an experimental approach was designed that would allow spatially resolved molecular characterisation of cellular neighbourhood and of the selected gene specific mutations (Supplementary Fig. 2).

### Identification of cellular neighbourhoods in colitis-associated neoplasia

**Figure 2:**
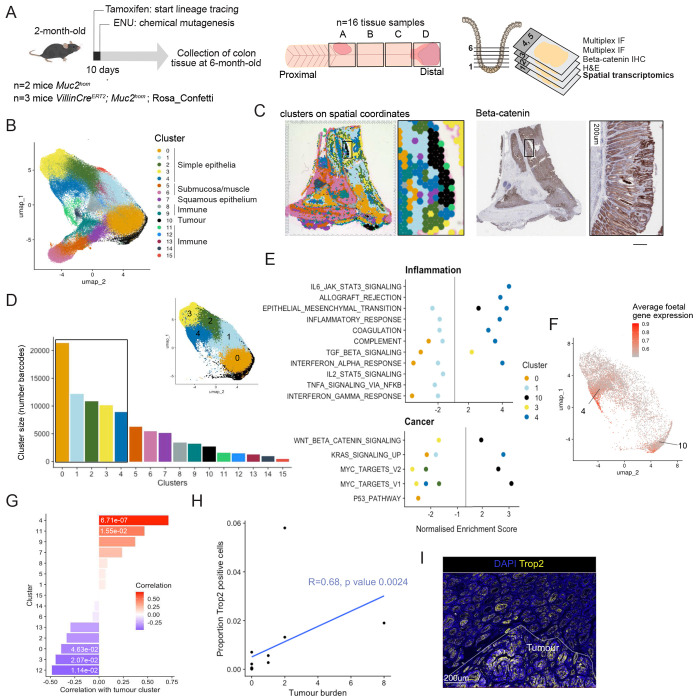
Epithelial neighbourhoods in inflammation-associated carcinogenesis. A) *Muc2*^*hom*^ mice received 1 IP injection of ENU at 8-weeks-old. 4 months post-ENU, proximal and distal pieces of colon tissue were collected and fixed. Serial sections were cut starting at the base of the crypt and going up and used for 1. spatial transcriptomic profiling with 10x Visium, 2. H&E staining, 3. IHC for beta-catenin, followed by multiplex immunofluorescence (n=5 mice, 16 tissue pieces). B) UMAP of clusters detected with Visium after integration of all slides. C) Clusters plotted on spatial coordinate and expression of beta-catenin on the same 11mm2 piece of *Muc2*^*hom*^ + ENU mouse colon. D) Bar plot depicting the number of Visium barcodes per cluster (inlet shows UMAP with main simple epithelial clusters 0-4 and tumour cluster 10. E) Results of GSEA showing significantly enriched pathways for each of the simple epithelial clusters, as well as the tumour cluster (p value < 0.05). F) Plot showing average expression of the foetal gene set^46^ on UMAP space. G) Bar plot showing correlation between coverage by each cluster and coverage by the tumour cluster (Kendall rank correlation coefficients on plot, p values < 0.05 annotated). H) Dot plot showing the relationship between tumour burden and number of Trop2-positive cells (n= 5 mice, 14 tissue samples, coefficient and p value for Kendall-rank correlation showed on plot). I) Representative immunofluorescence image showing a tumour arising in a Trop2-positive field.

First, to identify the cellular neighbourhoods present in chronic inflammation and associated neoplasia, we made use of the 10x Genomics Visium spatial transcriptomics protocol. Gene expression data was obtained from crypt-sized barcoded spots covering sixteen tissue samples from proximal and distal regions of the *Muc2*^hom^ mouse colon, which typically show the most advanced pathology^10^ (Fig. 2a). Gene expression data for all slides was integrated prior to unsupervised clustering and UMAP reduction (Supplementary Fig. 3a). Sixteen phenotypic clusters were identified and marker genes for each were found using differential gene expression analysis (Fig. 2b, Supplementary Fig. 3, Supplementary Table 1, Methods). Mapping spatial barcodes within each cluster to their spatial coordinates in tissue samples revealed that most clusters formed contiguous neighbourhoods (Supplementary Fig. 4a).

Several discrete clusters express immune cell markers, such as the B cell marker Cd19 ^21^ in cluster 14 or the neutrophil marker S100a9^22^ in cluster 11, indicating that they represent immune-enriched neighbourhoods (Supplementary Fig. 3b, c). Similarly, expression patterns of keratins were used to further inform the identity of intestinal epithelial neighbourhoods^23,24^ (Supplementary Fig. 3d). Widespread expression of keratin 8 indicates good epithelial representation across the dataset, with a gradient of keratin 20 suggesting higher colonocyte differentiation in clusters 2, 3 and 4 compared to clusters 0 and 1. Clusters 5 and 6 show abundant expression of tropomyosin, myopodin and actin, indicating that these neighbourhoods mainly comprise muscle cells. Keratin 14 is concentrated in cluster 7 and overlays squamous epithelium (Supplementary Fig. 3e). Cluster 10 shows high keratin 7 and low keratin 20 expression. Notably, keratin 7 is not expressed in healthy colonic epithelium but has been found elevated in IBD patients^25^, whilst low expression of keratin 20 indicates poor colonocyte differentiation. These observations, as well as β-catenin immunohistochemistry showing elevated nuclear β-catenin overlaying this cluster, identify it conclusively as the tumour cluster (Fig. 2c).

### Biological processes defining epithelial neigbhourhoods

To explore the biologically pathways active in the largest epithelial clusters and the tumour cluster, we performed GSEA using MSigDB hallmark pathways (Fig. 2d). Of note, genes were ranked based on differential gene expression in each cluster compared to all other clusters. As expected, the tumour cluster (cluster 10) showed enrichment for the Wnt and Myc pathways, as well as epithelial to mesenchymal transition (Fig. 2e). Markers for this cluster include Mmp7 and Lyz1, two genes normally associated with Paneth cells in a Wnt-high location in small intestinal crypts^26,27^ (Supplementary Fig. 3f, g). Interestingly, many genes marking the tumour cluster are related to the extracellular matrix (Supplementary Table 1), which has been described as a feature of nascent tumours^28^, indicating that we may be capturing early stages of neoplasia.

The five non-tumour simple epithelial neighbourhoods show varied responses to inflammation. Cluster 3 shows positive enrichment for TGFβ signalling and cluster 4 shows enrichment for many inflammatory pathways including interferon alpha (IFNα) as compared to other clusters. Cluster 4 also shows enrichment for coagulation markers, suggesting it might correspond to repairing epithelium. Accordingly, the average expression of foetal genes is higher in spots for which spatial barcodes map to cluster 4 (Fig. 2f). These results define cluster 4 as the neighbourhood associated with epithelial repair, and as the epithelial neighbourhood most responsive to inflammatory cues.

Clusters 0 and 1 show a drastically different response, with negative enrichment for many inflammation-related pathways such as interferon signalling, suggesting that cells in this neighbourhood are less responsive to immune activation (Fig. 2d). Interestingly, loss of function mutations in the gene *Nox1*, one of the top markers of cluster 0, has been described in several cases of very early onset IBD. Upregulation of this gene in cluster 0 could reflect a mechanism to prevent excessive inflammation.

### Evolution of cellular neighbourhoods with neoplasia

Cellular neighbourhoods evolve with the progression of disease, either shrinking or growing to occupy more of the tissue. To determine the dynamics of neighbourhoods that create an environment supporting tumour growth, we calculated the correlation between the proportion of spatial barcodes mapped to the tumour cluster within each tissue sample (coverage by the tumour cluster) and the coverage by each of the other clusters (Fig. 2g). The strongest positive correlation was seen between coverage with the tumour cluster and the tissue repair cluster 4 (R=0.71, p value 6.71e-05) (Fig. 2g), indicating a potential functional relationship between the establishment of a tissue repair environment and tumour formation. Accordingly, using immunofluorescence for the marker of tissue repair Trop2, we found a positive correlation between the proportion of Trop2 positive cells in a tissue sample and tumour burden (R=0.68, p value 0.0024) (Fig. 2h, i). Interestingly, both the tumour cluster 10 and tissue repair cluster 4 are co-occurring with the neutrophil cluster 11 (Cluster 10—11, R=0.47, p value 1.55e-02; Cluster 10—4, R=0.68 p value 2e-04) (Fig. 2g, Supplementary Fig. 4b). Neutrophil binding to ICAM-1 on epithelial cells was shown to trigger proliferation via Akt and β-catenin signalling^29^. In this context, it could enhance wound healing but could also drive Wnt-dependant neoplasia.

This analysis also revealed negative correlation between coverage of some clusters with the tumour cluster, indicating that those cellular neighbourhoods are shrinking with progression of neoplasia. This is the case for epithelial clusters 3 (Fig. 2g) (R=−0.45, p value 2.07e-02), which shows negative enrichment for the Myc pathway (Fig. 2e) and likely represents a healthy epithelial state. It is also the case for cluster 12 which is identified by the Iglc3 marker of plasma cells^30^ (R= 0.49, p value 1.14e-02). Infiltration with B and plasma cells has been associated with lower tumour stages and improved overall survival^31^. Interestingly, coverage by cluster 0 was also negatively correlated with coverage by the tumour cluster (Fig. 2g) (R=−0.39 p value 4.63e-02). This cluster corresponds to a cellular neighbourhood with dampened response to inflammation (Fig. 2e). Overall, this analysis revealed the dynamics of cellular neighbourhoods in the progression of disease, with response to inflammatory cues dictating association with neoplasia.

### Detection of mutations in known IBD and CAC genes

**Figure 3:**
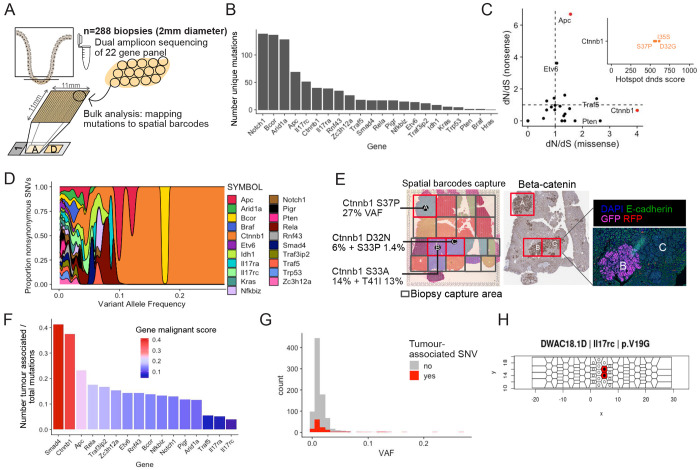
Epithelial mutational landscape in mutagen-treated chronically inflamed mouse colon. A) Tissue directly above the serial sections used for transcriptomic and multiplex IF profiling was sampled in series of 2mm diameter biopsies, to which was applied multiplexed targeted sequencing covering 22 genes previously identified in human IBD and/or CAC. B) Bar plot showing the number of mutations detected for genes covered in the panel (genes showed only if mutations were detected). C) Dot plot showing selection for nonsense mutations (y-axis) and missense mutations (x-axis). Red dots denote significant positive selection (dN/dS analysis, see methods). Inlet shows results from dN/dS hotspot analysis. D) Density plot showing proportion of nonsynonymous and stop-gained SNVs at different Variant Allele Frequencies (VAF). E) Grid and colours depict biopsies used for targeted amplicon sequencing (3 biopsies are highlighted in red and SNVs in Ctnnb1 annotated with VAF). The same biopsies are represented on a Beta-catenin IHC, and on the right the corresponding immunofluorescence image shows lineage tracing with GFP detecting one clone corresponding to tumour B. F) Bar plot showing number of tumour-associated / total nonsynonymous mutations per gene, representing the malignant potential of each gene. G) Histogram showing VAF distribution for tumour-associated SNVs (red) or non-tumour-associated SNVs (grey). H) Tessellation mask for a piece of tissue showing expansion of an Il17rc mutated non-malignant clone.

To determine whether the accumulation and expansion of mutations found in human IBD and associated cancer is linked with specific epithelial neighbourhoods, we profiled the mutational landscape in the colonic epithelium of the same cohort of *Muc2^KO^* mice treated with ENU. Colon tissue directly overlaying the sections used for spatial transcriptomics and immunofluorescence profiling was extensively sub-sampled in a series of 2mm diameter biopsies (Fig. 3a). Replicate targeted-amplicon sequencing for 22 genes commonly found mutated in IBD and/or CAC (Supplementary Table 2) was applied to each of the 288 biopsies. To correctly assign mutations as clonal events, spatial coordinates were used to detect adjacent biopsies with the same mutation (Supplementary Fig. 5 and Methods). Single Nucleotide Variants (SNVs) were detected in all genes covered, including known CAC drivers *Trp53*, *Kras* and *Smad4*^4^, but also several genes associated with the IL-17 pathway (*Il17rc*, *Il17ra*, *Pigr*) and chromatin remodellers *Arid1a* and *Bcor* commonly found mutated in the non-dysplastic colonic epithelium of IBD patients^1–3^ (Fig. 3b, Supplementary Table 3).

Analysis of dN/dS ratios identified *Ctnnb1* and *Apc* as genes under significant positive selection for missense and nonsense mutations, respectively (Fig. 3c). This selection is also reflected in the variant allele frequencies (VAFs) reached by *Ctnnb1* mutations, which dominate the SNV landscape above 10% VAF (fig. 3d). Such mutations could be mapped to tumours with elevated nuclear β-catenin staining, with VAFs reaching up to 27% in a single biopsy (Fig. 3e). Together, this suggests a dependence on Wnt signalling for tumour growth.

### Paired transcriptional profiling and mutation calling identifies tumour-associated mutations

To define the potential for each mutation to drive malignant transformation in the model, we leveraged transcriptomic information obtained from the overlying tissue samples by extracting spatial barcodes corresponding to each biopsy to enable a pseudo-bulk analysis (Supplementary Fig. 2, Fig. 3a). The proportion of spatial barcodes mapping to each epithelial cluster was obtained for the 232 biopsies captured in the Visium experiment. Tumour-associated mutations were defined as any non-synonymous SNV detected in a biopsy containing at least 5 spatial barcodes mapping to the tumour cluster (Supplementary Fig. 6a, Supplementary Table 4).

The proportion of tumour-associated to total non-synonymous mutations was used to derive a malignancy score representing the malignant potential of each gene (Fig. 3f). This revealed *Smad4* and *Ctnnb1* as the genes most associated with malignancy (41% and 37% malignancy scores, respectively), as opposed to inflammatory genes (e.g. 13% and 4% for *Nfkbiz* and *Il17rc*, respectively). Consistent with the dN/dS analysis, amino-acid positions 32-41 were identified as a hotspot in the *Ctnnb1* gene relating to oncogenic activity arising from stabilisation of the protein (Supplementary Fig. 6b). Although *Ctnnb1* mutations are uncommon in human colon cancer, *Smad4* mutations are associated with poor-prognosis, recurrence and resistance to treatment in colorectal cancer patients^32,33^ and found mutated in 13% of CACs^4^. The most mutated *SMAD4* position in human colon cancer is 361, with substitution from an arginine to a histidine (p.R361H). Here, tumour-associated *Smad4* mutations include 6 missense mutations at positions 160, 194, 294, 307, 360 and 376, and a nonsense mutation at position 194 (Supplementary Table 4).

### Expansion capability of tumour- and non-tumour-associated mutations

To determine the capacity of tumour- and non-tumour-associated mutations to expand, the VAF was used as a surrogate of mutated clone size (Fig. 3g). As expected from the VAF distribution of Ctnnb1 mutations (Fig. 3d) and the high malignancy score of this gene (Fig. 3f), tumour-associated SNVs reached high VAFs corresponding to large tumours. In accordance, half of clones spanning more than one biopsy correspond to missense mutations in Ctnnb1, followed by a quarter of *Apc* truncating mutations (Supplementary Fig. 5), largely reflecting the size which can be reached by clones after malignant transformation. Only a single large expansion detected in *Il17rc* was not tumour-associated (Fig. 3h). Below 5% VAF however, the distribution of clone size for either tumour- or non-tumour-associated mutations is similar (Fig. 3g). Furthermore, clones bearing mutations that have not been linked with neoplasia can expand to reach more than 5% VAF in a single biopsy. This is the case for Arid1a p.Y473*, Il17ra p.I405V, Nfkbiz p. M414K and Rela p.Y66C (Supplementary Table 3). This indicates that prior to neoplastic transformation, clones bearing either tumour- or non-tumour-associated mutations have similar expansion capability.

### Tissue repair promotes expansion of pro-oncogenic clones

**Figure 4:**
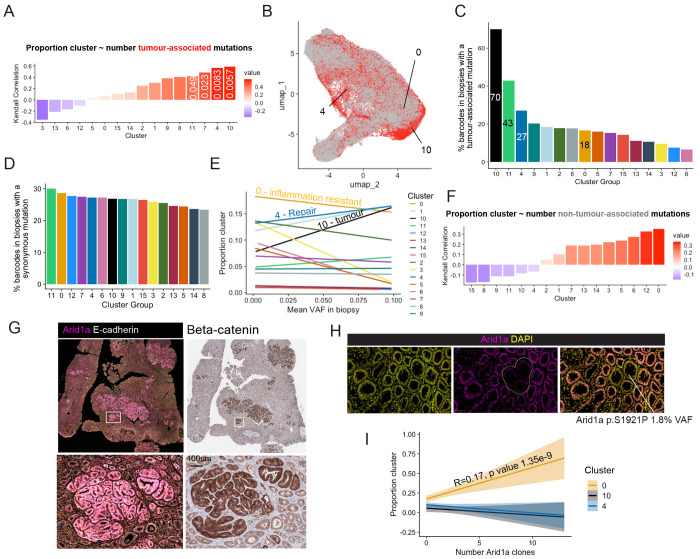
Selection of malignant and non-malignant clones in epithelial neighbourhoods. A) Plot showing correlation between number of tumour-associated mutations in each biopsy and the proportion of spatial barcodes mapped to each cluster (Kendall-ranked coefficient showed on plot, p values < 0.05 annotated. B) UMAP of spatial barcodes that could be mapped to biopsies, red colour showing barcodes mapped to biopsies containing a tumour-associated mutation. C) Bar plot showing percentage of spatial barcodes mapped to a biopsy containing a tumour-associated mutation for each cluster. D) Bar plot showing percentage of spatial barcodes mapped to a biopsy containing a synonymous mutation for each cluster. E) Line plot showing the linear relationship between mean VAF in a biopsy and proportion of each cluster (Number and name of clusters of interest annotated). F) Plot showing trends in correlation between number of non-tumour-associated mutations in each biopsy and the proportion of spatial barcodes mapped to each cluster (Kendall-rank test coefficients on plot). G) Immunofluorescence images stained for Arid1a (left) and associated beta-catenin staining (right) showing up-regulated Arid1a protein expression in tumours. H) Immunofluorescence images stained for Arid1a, with DAPI nuclear counterstain, showing loss of Arid1a staining corresponding to an Arid1a missense mutation detected at 1.8% VAF. I) Line plot showing the linear relationship between number of Arid1a-depleted clones detected with immunostaining in a biopsy and proportion of each cluster (standard error shaded, global linear model estimate and p value showed on plot when p value < 0.05).

To determine whether preferential selection of tumour-associated mutations could explain the relationship identified between the tissue repair neighbourhood defined by cluster 4 and the tumour cluster, we examined the correlation between the number of tumour-associated mutations and the proportion of tissue covered by each cluster (fig. 4a). This revealed a positive correlation for the tissue repair cluster 4 (Kendall-rank test correlation coefficient *R* = 0.56, p value 8.3e-03). This association was tested by mapping tumour-associated mutations to the UMAP space (Fig. 4b), revealing a 27% probability for a spatial barcode in a biopsy holding a tumour-associated mutation to fall within the epithelial repair cluster 4, second only to the tumour and neutrophil clusters (Fig 4c). In contrast and as expected, spatial barcodes in biopsies containing synonymous SNV have an equal chance to be part of any cluster (Fig. 4d). This suggests that compared to neutral events, pro-oncogenic clones preferentially appear in a tissue repair context. Furthermore, below 10%, VAFs for tumour-associated mutations increase with the proportion of the tissue sample covered by the epithelial repair neighbourhoods (Global linear model intercept = 0.13 p value = 1.59e-6) (Fig. 4e). The preferential emergence of tumour-associated mutations in the context of tissue repair provides a mechanistic link explaining why tumours are initiated in repairing epithelium in the context of chronic inflammation.

### Non-oncogenic clones accumulate in immune resistant neighbourhoods

Importantly, the correlation between the number of non-tumour-associated mutations and proportion of the tissue sample covered by the immune resistant cluster 0 was the highest of all clusters (R= 0.35, p value 0.066) (Fig. 4f). Furthermore, the chance for a spatial barcode in a biopsy holding a tumour-associated mutation to fall in the inflammation resistant epithelial cluster 0 is the lowest out of all epithelial cluster except for the healthy cluster 3 (Fig. 4c), To determine whether the inflammation-resistant environment of cluster 0 could promote expansion of non-oncogenic clones, we used immunostaining to detect variation in expression of the *Arid1a* gene. Deletion of this gene is usually presented as a driver of colorectal cancer, however this gene is also highly mutated in non-dysplastic IBD epithelium^1–3^. Here, despite a high mutation burden, the malignant score of Arid1a is only 12%, suggesting that most mutations in this gene do not drive tumorigenesis. Immune exclusion has recently been observed in Arid1a-mutant clones in normal human colon epithelium^34^ and could explain why mutations in this chromatin remodeller shows a similar profile to mutations in inflammatory genes. Using immunostaining, we could detect Arid1a upregulation at the protein level in tumours (Fig, 4g), suggesting that non-mutational stabilisation of Arid1a could be required in cancer cells. Such processes have been described in liver tumorigenesis, another inflammation-driver cancer^35^. The same antibody allowed the detection of loss or downregulated expression of Arid1a in mutant clones (Fig. 4h). We find that the number of Arid1a-deficient clones detected through immunostaining increases with the proportion of the biopsy covered by the immune resistant cluster 0 (Global linear model intercept = 0.17 p value = 1.35e-9) (Fig. 4i), confirming a strong association between presence of mutations in genes that have low malignancy score and the establishment of environments where response to inflammatory cues is dampened.

## DISCUSSION

Mutant clones selected in IBD may impact pathogenesis and development of colitis associated cancer. Detailed cataloguing of mutations, assessment of their location and function in patients is made complicated by the rarity of such events, sampling methods and confounding effects of treatment. Here, a mouse model of colitis at early stages of tumour initiation was leveraged to profile the mutations selected and the environment they arise in, which together shape the conditions favouring different phenotypic outcomes, including neoplasia. Importantly, a framework for combined detection of somatic mutations and profiling of associated changes in gene expression is provided.

Tissue repair and the associated remodelling of the extracellular matrix has been described as cancer promoting^36–38^. Here, we find a positive correlation between establishment of a repairing epithelial environment, accumulation and expansion of pro-oncogenic mutations, and eventual malignant transformation. Therefore, altough tissue repair is a wanted outcome in chronic inflammatory diseases, the risk of neoplastic transformation in such contexts must be considered. Here, the process by which epithelial repair promotes clonal expansion by crypt fission is outlined. This process is neutral, but mutations conferring an increased fission capacity gain a significant advantage. Arid1a-mutated clones have been shown to display fission bias^34^, which may explain their presence in CAC samples despite the low malignant score found for this gene in our study.

In contrast, the establishment of an inflammation-resistant environment was associated with accumulation of non-oncogenic clones previously found in the non-dysplastic epithelium of IBD patients^1–3^. The extent of expansion of such mutations in normal tissue both in this study and previous work in patients samples suggests they may be contributing to the maintainance of such inflammation-resistant environment. Despite evidence from previous studies pointing to a role in worsening the activity of Ulcerative Colitis, an implication of our work is that establishement of an inflammation-resistant environment may act to prevent expansion of pro-oncogenic mutations, thereby delaying malignant transformation. Notch1 clonal expansion has been described as anti-oncogenic in the context of the oesophagus, where such clones compete with cancer-drivers to prevent their transformation^39,40^. Further studies will need to address the functional link between expansion of mutations in inflammatory and chromatin remodelling genes and competition with pro-oncogenic mutations. Promoting tissue repair whilst controlling malignant transformation is of upmost importance for long-term management of IBD, and particularly revelant considering recent epidemiology studies showing rising incidence of IBD in children, with a decrease of the childhood age of onset^41^.

## METHODS

### Mice

Mice were of C57BL/6 background. The *Muc2^KO^* line used was described by Velcich *et al.*^11.^ Mice containing inter-crossed *R26^Confetti^* and *VillinCre^ERt2^* alleles^12^ were bred to *Muc2^KO^* mice to generate *VillinCreERt2; Muc2^KO^; R26^Confetti^* mice. Genotyping was outsourced to Transnetyx (Cordova, TN, USA).

### Animal husbandry

Animal care and procedures were performed at the Cancer Research UK Cambridge Institute Biological Resource Unit according to the UK Home Office under the authority of a Home Office project licence (PD5F099BE) approved by the Animal Welfare and Ethical Review Body at the CRUK Cambridge Institute, University of Cambridge. Mice were housed under controlled conditions (temperature (21 ± 2°C), humidity (55 ± 10%), 12h light/dark cycle) in a specific-pathogen-free (SPF) facility (tested according to the recommendations for health monitoring by the Federation of European Laboratory Animal Science Associations). Animals had unrestricted access to food and water. None of the mice had been involved in any procedure prior to the study

### 3D imaging and clone counting

The whole colon of *VillinCreERt2; Muc2^KO^; R26^Confetti^* mice was dissected, flushed with cold PBS, cut longitudinally, and whole-mounted. Following fixation in 4% paraformaldehyde overnight at 4 °C, the tissue was washed in PBS and selected colon segments were excised. Optical clearing was performed using the CUBIC protocol^42^. In brief, excised segments were incubated with CUBIC-1a solution (10% urea, 5% *N*,*N*,*N*′,*N*′-tetrakis(2-hydroxypropyl) ethyl-enediamine, 10% Triton X-100 and 25 mM NaCl in distilled water) at 37 °C for 7–10 days with alternate day solution changes. DAPI was used for nuclear counterstaining at a dilution of 1:1,000. The cleared tissue was then washed in PBS for 24 h. Additional clearing and refractive index matching were performed with Rapiclear 1.52 (152002, SunJin Labs) for 24 h. Samples were mounted in a 0.25 mm i-Spacer (Sunjin Labs) for confocal imaging on a Leica SP5 TCS confocal microscope (LAS software v2.8.0, Leica Biosystems) with a 10× objective, 1.4–1.7 optical zoom and 8–12 μm *z*-steps throughout the whole thickness of the tissue. Clone counting was performed using the cell counter tool in the ImageJ software.

### Immunohistochemistry

Mouse colons were opened longitudinally and cut in approximately 15mm^2^ sections placed in cassettes and fixed overnight at 4 °C in 4% paraformaldehyde. Haematoxylin and eosin (H&E) staining was performed using an automated ST5020 Multistainer (Leica Biosystems). Staining for β-catenin was performed on Leica’s automated Bond-III platform in conjunction with the Polymer Refine Detection System (DS9800, Leica Biosystems). In brief, epitope retrieval was performed using Leica Epitope Retrieval Solution 1 (AR9961, Leica Biosystems) at 100 °C. Blocking was performed with Protein Block Buffer (X090930-2, Dako). Following incubation with primary antibody against β-catenin (0.25 μg ml^−1^, mouse, 610154, BD Biosciences), sections were incubated with secondary antibody (1:1,500, rabbit anti-mouse IgG1, ab125913, Abcam) before development and mounting. For β-catenin staining, an additional mouse-on-mouse blocking step was performed.

### Immunofluorescence

Heat-mediated epitope retrieval was performed on rehydrated 3-μm-thick paraffin sections in 10 mM sodium citrate (pH 6.0). The sections were then incubated in blocking solution (10% donkey serum and 0.05% Tween-20 in PBS) at room temperature for 30 min. Primary antibodies against RFP (1:100, rabbit, R10367, Thermo Fisher), GFP (1:100, chicken, ab13970, Abcam), E-cadherin (1:200, mouse, 610182, BD Biosciences), Trop2 (1:100, goat, AF1122, R&D systems) and Arid1a (1:100, rabbit, 12354S, Cell Signalling) were diluted in blocking solution, in which sections were then incubated in the dark overnight at 4 °C. Sections were washed and incubated with fluorophore-conjugated secondary antibodies (donkey anti-rabbit A31572, donkey anti-goat A21447 and/or donkey anti-chicken 703-6-5-155, Thermofisher and/or donkey anti-mouse ab150109, Abcam) diluted 1:200 in 0.05% Tween-20 in PBS for 45 min at room temperature. DAPI (1:1,000) was used for nuclear counterstaining. After washing, the stained sections were mounted using ProLong Gold Antifade Mountant (P36930, Thermo Fisher,).

### RNAscope

Simultaneous detection of *Notum* and *Reg4* was performed on paraffin embedded sections using Advanced Cell Diagnostics (ACD) RNAscope 2.5 LS Duplex Reagent Kit (322440), RNAscope 2.5 LS Probe- Mm- Notum C1 (428988), and RNAscope 2.5 LS Probe-Mm-Reg4-C2 (409608). Three-micrometre-thick sections were baked for 1 h at 60 °C before loading onto a Bond RX instrument (Leica Biosystems). Slides were deparaffinized and rehydrated on board before pre-treatments using Epitope Retrieval Solution 2 (AR9640, Leica Biosystems) at 95 °C for 15 min, and ACD Enzyme from the Duplex Reagent kit at 40 °C for 15 min. Probe hybridization and signal amplification were performed according to manufacturer’s instructions. Fast red detection of C2 was performed on the Bond Rx using the Bond Polymer Refine Red Detection Kit (DS9390, Leica Biosystems) according to ACD protocol. Slides were then removed from the Bond Rx and detection of the C1 signal was performed using the RNAscope 2.5 LS Green Accessory Pack (322550, ACD) according to kit instructions. Slides were heated at 60 °C for 1 h, dipped in Xylene and mounted using VectaMount Permanent Mounting Medium (H-5000, Vector Laboratories). The slides were imaged on the Aperio AT2 (Leica Biosystems) to create whole slide images. Images were captured at 40× magnification, with a resolution of 0.25 μm per pixel.

### Inference of effective fission rate from lineage tracing data

The statistical model for crypt fission described in Nicholson *et al.*, 2018 and implemented in the R package RHClones (https://github.com/ElEd2/RHClones) was adapted to infer effective fission rates using manual counts of clone sizes from tissue regions in *Muc2*^het^ and *Muc2*^hom^ mice with different average *Trop2* expression levels. In brief, each tissue region was assigned a local fission rate $\rho_{i}$ (units 1/days) that is sampled from a hierarchical Student’s T prior

$$\rho_{i}∼StudentT(\nu ,\mu ,\sigma )$$

with population-level parameters

$$\rho ∼Normal(0,10^{-2})\;\sigma ∼Normal(0,10^{-2})\;v∼Gamma(2,10^{-1}).$$

The vector of observed clone sizes, $g_{i}$, for region i is then distributed according to

$$g_{i}∼Multinomial(f(\rho_{i},t_{i}))$$

where

$$f_{n}(\rho ,t)=e^{-\rho t}{\left(1-e^{-\rho t}\right)}^{n-1}$$

is the solution of the Yule-Furry pure birth process, and $t_{i}$ is the time (in days) since clone induction in region i.

The model for fission inference in this study differs from the model for fission after continuous clone induction described in Nicholson *et al.*, 2018 where this solution is integrated over the age of the individual. However, as in the aforementioned study, a correction was applied to $f_{1}$ and $f_{2}$ to correct for the chance occurrence of neighbouring, non-clonally related crypts. This model does not incorporate crypt fusion, nor does it account for tissue re-modelling that may partly explain clone sizes in tissue regions associated with repair following inflammation. In this respect, inferred fission rates should be interpreted as “effective” in the sense that they only capture the value of $\rho_{i}$ that is required to explain clone sizes using the Yule-Furry pure birth process introduced in Nicholson *et al.*, 2018.

### Simulations of crypt dynamics

Crypt dynamics were simulated using an on-lattice, voter-type model. The square lattice is of shape M×M. For a site (i,j) on the lattice, the neighbouring sites are defined to be the set:

$$N_{i,j}=\{(i,j+1),(i,j-1),(i+1,j),(i-1,j),(i+1,j+1),(i-1,j-1)\},$$

resembling a configuration equivalent to a hexagonal lattice in two dimensions with periodic boundary conditions. The state of a lattice site (i,j), denoted by $s_{i,j}$, is defined as $s_{i,j}=-1$ for unlabelled (UL) sites, $s_{i,j}=0$ for empty sites and $s_{i,j}=+1$ for labelled (L) sites. Assuming a labelling efficiency of 20%, the initial population on the lattice consists of 80% of UL and 20% of L sites randomly assigned to each site. The parameters governing the simulations are the fission $\left(\rho_{s}\right)$, fusion $\left(f_{s}\right)$ and fixation $\left(P_{s}\right)$ probabilities of the state s=±1. All are assumed to be neutral and equal for L and UL crypts as the baseline scenario ($\rho_{s},f_{s},P_{s}=0.5$ for s=±1, undefined otherwise). To determine the impact of fission bias in tissue repair, the fission probability of L sites was increased to $\rho_{1}=0.95$, with all other parameters remaining the same.

The simulation rules are that, at each time step, we choose a random site (i,j) and update the lattice state according to:

1. If (i,j) is an empty site $\left(s_{i,j}=0\right)$:
  - and all neighbours are empty sites ($s_{k,l}=0\forall (k,l)\in N_{i,j}$), do nothing;
  - otherwise, choose a non-empty neighbour site $(k,l)\in N_{i,j}:s_{k,l}\neq 0$ at random to undergo fission by setting $s_{i,j}=s_{k,l}$ with probability $\rho_{s_{k,l}}$ (Supplementary Fig. 2a).
2. If (i,j) is non-empty $\left(s_{i,j}\neq 0\right)$, then choose a neighbour $(k,l)\in N_{i,j}$ at random and:
  - if (k,l) is empty then (i,j) undergoes fission by setting $s_{k,l}=s_{i,j}$ with probability $\rho_{s_{i,j}};$
  - otherwise, fusion occurs with probability $\frac{f_{s_{i,j}}+f_{s_{k,l}}}{2}$ by choosing (h,w)∈{(i,j),(k,l)} with equal probability and setting $s_{h,w}=0$. The outcome of monoclonal fixation following fusion is then determined to be the state $s^{\prime }$ according to the fixation probabilities $P_{s_{i,j}}$ and $P_{s_{k,l}}$ as outlined in Supplementary Fig. 2b and 2c and then setting $s_{p,q}=s^{\prime }$ where (p,q)∈{(i,j),(k,l)}∖{(h,w)}.

We note that our definition of homeostasis in *in silico* models is not equivalent to an equilibrium since, while the average numbers of labelled and unlabelled crypts remain constant, their spatial distribution across the lattice undergoes domain coarsening as captured by a “site aggregation” metric that slowly increases across the course of simulations (Methods and Figure 1f, l). Furthermore, fission biased conditions lead to a non-homeostatic system even in the absence of damage, since higher expansion rates generally outpace the creation of empty space by crypt fusion. This is reminiscent of a regime where additional mechanisms, such as diffusion (not considered here), may be required to spatially accommodate mutant crypts with higher rates of fission (Olpe et al., 2021).

### Calculation of site aggregation

The number of distinct neighbour pairs (DNPs) was quantified to represent aggregation of labelled and unlabelled sites in the lattice. The number of the DNPs for a (i,j) on the lattice is defined to be the *cardinality* of the set:

$$G_{i,j}=\left\{\left(s_{i,j},s_{k,l}\right):(k,l)\in N_{i,j},s_{i,j}\neq s_{k,l},s_{i,j}\neq 0,s_{k,l}\neq 0\right\}.$$

The neighbour pairs (NPs) of a site (i,j) is the set:

$$K_{i,j}=\left\{\left(s_{i,j},s_{k,l}\right):(k,l)\in N_{i,j},s_{i,j}\neq 0,s_{k,l}\neq 0\right\}.$$

To obtain a measure of DNPs independent of grid size, we consider the concentration of DNPs, defined as:

$$C=\frac{\alpha }{\beta },$$

where α and β are the total number of DNPs and NPs over the whole lattice, respectively. Importantly, we considered aggregation of labelled and unlabelled sites, excluding empty sites, and plotted site aggregation as 1−C, so that increasing values denote a decreasing concentration of DNPs.

### Bulk RNA sequencing

RNA was collected from n=3 *Muc2^hom^* and 3 *Muc2^het^* mouse colons from 10-months-old littermates. mRNA extraction was performed following instructions from the extraction kit (180244, Qiagen). Library prep was performed by the CRUK Cambridge Institute Genomics Core using the Illumina Stranded mRNA Prep kit (20040532, Illumina) according to manufacturer’s instructions. Samples were sequenced using the Illumina Novaseq platform with 50 bp paired end reads. Differential expression analysis was performed using DESeq2. Expressed genes were ranked by descending log2 fold change for use in gene set enrichment analysis (Subramanian et al., 2005).

### Setup for paired Spatial Transcriptomic and DNA sequencing

A cohort of five *Muc2*^hom^ mice were treated with ENU 8 weeks post birth and aged for 16 more weeks before collection. Whole-mounted colons were fixed, and 6 serial tissue sections of 3-5μm thickness were cut at the crypt base, for at least three areas per colon representing varied levels of pathology. Levels were used for 1. Spatial transcriptomics profiling, 2. Pathology assessment with H&E, 3. Tumour counting with Beta-catenin immunohistochemistry, 4. Clone counting with GFP, RFP + DAPI and E-cadherin counterstain), 5. Arid1a-mutated clone counting with Arid1a + DAPI counterstain.

### Spatial Transcriptomics

Slide processing and library preparation were performed according to the 10X Visium Cytassist FFPE protocol. Briefly, 5μm sections of tissue were transferred to fit each of the two 11m2 oligo-barcoded capture areas on Visium 10x Genomics slides. Libraries were processed by the CRUK Cambridge Institute Genomics Core according to the manufacturer’s instructions and sequenced on Illumina’s NovaSeqX Plus to an average depth of 8 million mapped reads per sample. Fastq files were processed using the Spaceranger command line tool (10x Genomics v3.0.1) and mapped to the pre-built mm10 reference genome. Processed gene expression matrices for each slide were converted to a Seurat object. After normalisation by variance stabilising transformation using SCTransform, objects corresponding to each slide were integrated into a merged Seurat object using Harmony^43^. Clusters were identified using FindNeighbors using integration anchors at a UMAP dimension of 0.5. Genes differentially expressed in each cluster were derived using FindMarkers. Top markers for each cluster indicated in Supplementary Fig. 2b were selected based on log2 fold change showing high expression in the cluster, high pct.1 showing good representation in spatial barcodes mapped to the cluster and low pct.2 showing poor representation in spatial barcodes mapped to other clusters. For pseudo-bulk analysis, LoupeBrowser was used to extract spatial barcodes assigned to each of the 2mm-diameter biopsies from which DNA was sequenced.

### NGS library preparation with Targeted DNA sequencing library assays

Genes of interest were imported in the Fluidigm D3 Assay design platform and dual coverage primers were designed (Supplementary Table 5). 8 amplicons pools were prepared and used in the LP 8.8.6 integrated fluidic circuit (IFC) (101-7663) according to manufacturer’s instructions. Samples were sequenced in 2 separate runs by the CRUK Cambridge Institute Genomics Core on Illumina NovaSeqX Plus for 150bp paired end reads.

### Mutation calling

RePlow was used for mutation calling based on dual replicate amplicon coverage^44^ using combinatorial pooling of amplicons. In brief, RePlow exploits replicate library preparations to separate the contribution of background errors occurring in library preparation from sequencing errors occurring in sequencing. This is done with a statistical model that calculates the total log ratio of probabilities for variant candidates compared to background errors across all replicates simultaneously. In doing so, adjusted variant allele frequencies (VAFs) are constructed by subtracting the contribution from sequencing errors, while background error profiles for the error model are calculated independently for the six base pair substitution types (A>C, A>G, A>T, C>A, C>G, C>T) across the targeted regions. An additional stringent filtering step was applied whereby only variants with a positive log ratio of probabilities in both replicates individually were retained, to exclude false positive calls at low VAF values. Orthogonal validation of mutation calls was done using the Ampliconseq pipeline (https://github.com/crukci-bioinformatics/ampliconseq). Briefly, variants are called using Vardict from sequence reads aligned to the reference genome (GRCm39). The pipeline models the background substitution noise at each amplicon position to identify and filter SNV calls with an allele fraction indistinguishable from noise. A minimum of 0.01% VAF was set as the lower detection threshold. Outliers in the VAF/noise threshold plane, corresponding to remaining artefacts and germline SNVs were removed post-hoc.

### Tessellation algorithm for clone calling

To parsimoniously assign multiple calls of the same mutation made from the same piece of colon tissue to individual clones, as well as resolve the spatial context of the colon that was opened longitudinally prior to biopsy sampling, a Voronoi tessellation with periodic boundary conditions along the radial axis of each tissue section was calculated using the spatial coordinates of the corresponding biopsies. A depth-first-search algorithm was then employed to call single clones by enumerating all connected components of the graph with edges defined by adjacent Voronoi tiles (or those found within 2mm from one another) containing the given mutation.

### Quantifying selection using dN/dS

The latest version of the maximum likelihood implementation of dN/dS, as origionally described in Martincorena *et al.*^45^ and available in the *R* package dNdScv (https://github.com/im3sanger/dndscv), was applied to identify genes under positive selection. A custom reference object was built from the assembly GRCm39 by sub-setting the trinucleotide context-dependent substitution consequence matrix to the set of possible mutations based on the region of the genome selected for targeted sequencing in this study. Counts of mutations fed into the algorithm were those based on individual clones, as defined above.

### Statistical analyses and reproducibility

Visualization and statistical analysis of data were performed in the R statistical computing environment (version 2024.04.0+735). All experiments were performed on at least three independent biological replicates (three different mice). Micrographs depict representative data derived from at least three independent biological replicates. Normality was assessed using Shapiro-Wilk’s tests and relevant statistical tests applied. Tests and corresponding *P* values are indicated in the figure legends and figures, respectively. Box plots display the distribution of data using the following components: lower whisker show the smallest observation greater than or equal to lower hinge minus 1.5× IQR; lower hinge shows the 25% quantile; the centre line shows the median, 50% quantile; the upper hinge shows the 75% quantile; the upper whisker shows the largest observation less than or equal to upper hinge plus 1.5× IQR.

## Supplementary Material

1
