# Supplementary material for: Paired mutation calling and spatial transcriptomics identify cellular neighbourhoods dictating the neoplastic outcome of colitis
Source: Res Sq. 2025 May 8:rs.3.rs-6401505. Preprint. [Version 1] doi: 10.21203/rs.3.rs-6401505/v1 (PMC12083657; doi:10.21203/rs.3.rs-6401505/v1)
Supplement: 1 [file NIHPPrs6401505v1-supplement-1.pdf]

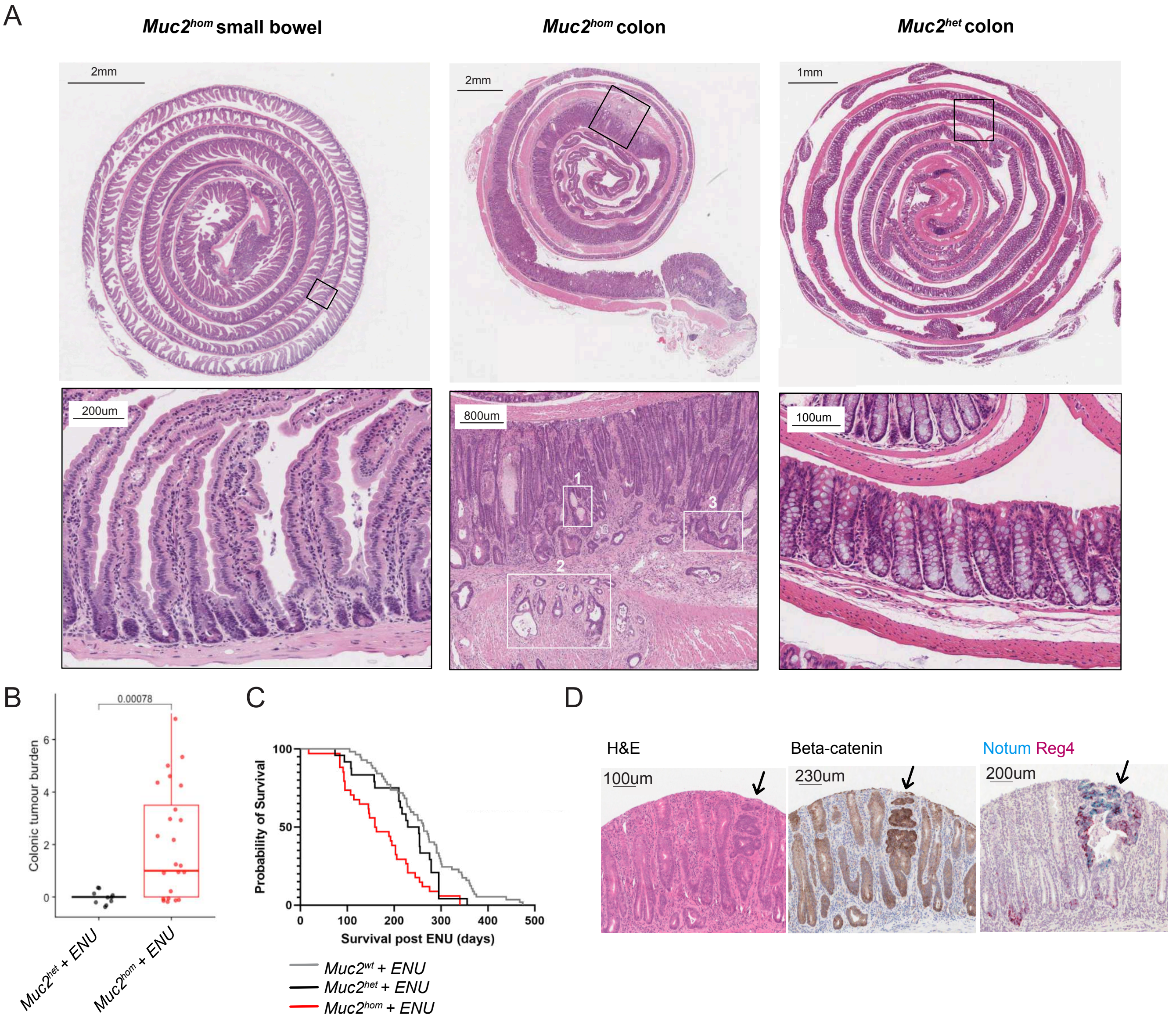

Supplementary Figure 1: Modelling colitis and associated-cancer A) Swiss-rolled *Muc2^hom^* small bowel, *Muc2^hom^* colon and *Muc2^het^* colon stained with haematoxylin and eosin (H&E). Panel on the bottom zooms in representative areas and reveals signs of pathology in *Muc2^hom^* colon (1. abscess, 2. epithelial foci invading muscle layer, 3. loss of crypt polarity). B) Number of colonic nuclear beta-catenin tumours found in a cohort of n=23 *Muc2^hom^* and n=11 *Muc2^het^* mice (p value for Wilcoxon signed rank-test showed on graph). C) Survival post-ENU for wild-type, *Muc2^hom^* and *Muc2^het^* mice (n=57, 34 and 24 respectively). D) Images showing H&E staining, beta-catenin IHC and Notum/ Reg4 RNAscope on an on-edge piece of tissue from the colon of a *Muc2^hom^* mouse treated with ENU. Arrow points to a tumour.

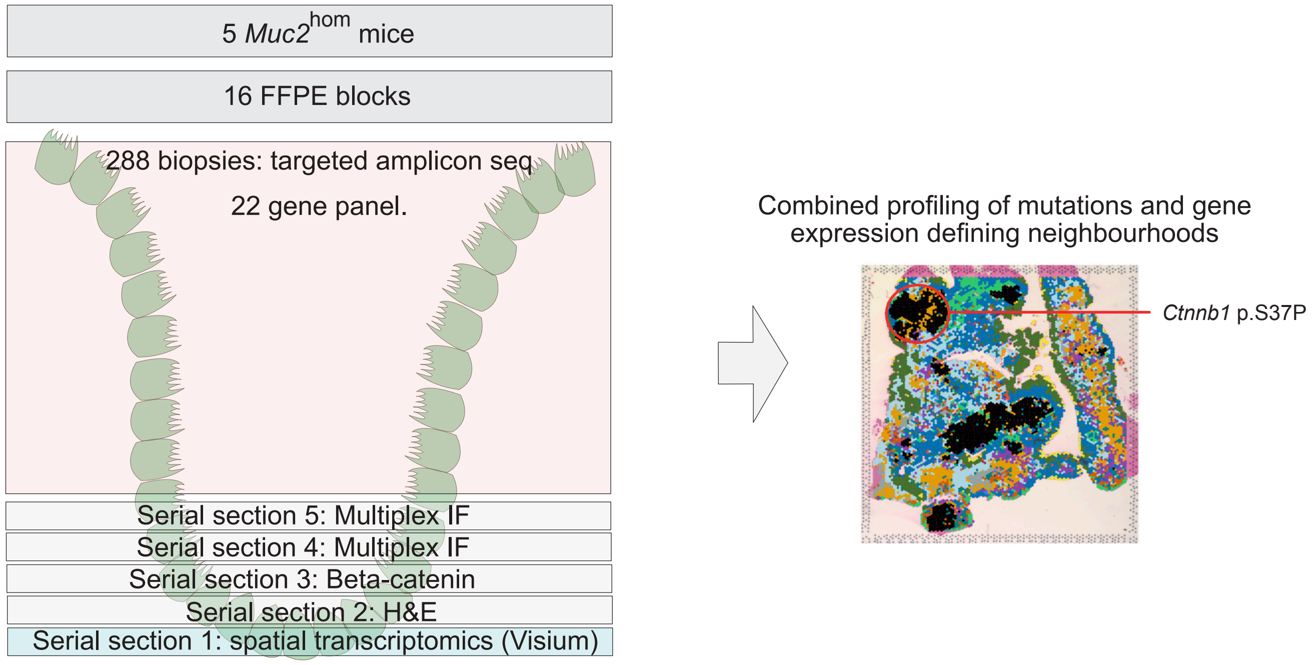

Supplementary Figure 2: Approach for joint mutation calling and profiling of cellular neighbourhoods. 16 FFPE colon tissue samples were obtained from 5 Muc2hom mice treated with ENU. Serial sections were cut from the base of the crypts towards the lumen and used for 1. spatial transcriptomic profiling with the 10x Visium FFPE protocol, 2. Haematoxylin and Eosin (H&E) staining, 3. Immunohistochemistry (IHC) for beta-catenin, followed by multiplex immunofluorescence (IF) (4. E-cadherin, GFP, RFP, Trop2, 5. DAPI, E-cadherin, Arid1a) (n=5 mice, 16 tissue pieces). Tissue directly above the serial sections used for spatial transcriptomic and multiplex IFs was sampled in series of 2mm diameter biopsies, to which was applied multiplexed targeted sequencing covering 22 genes previously identified in human IBD and/or CAC.

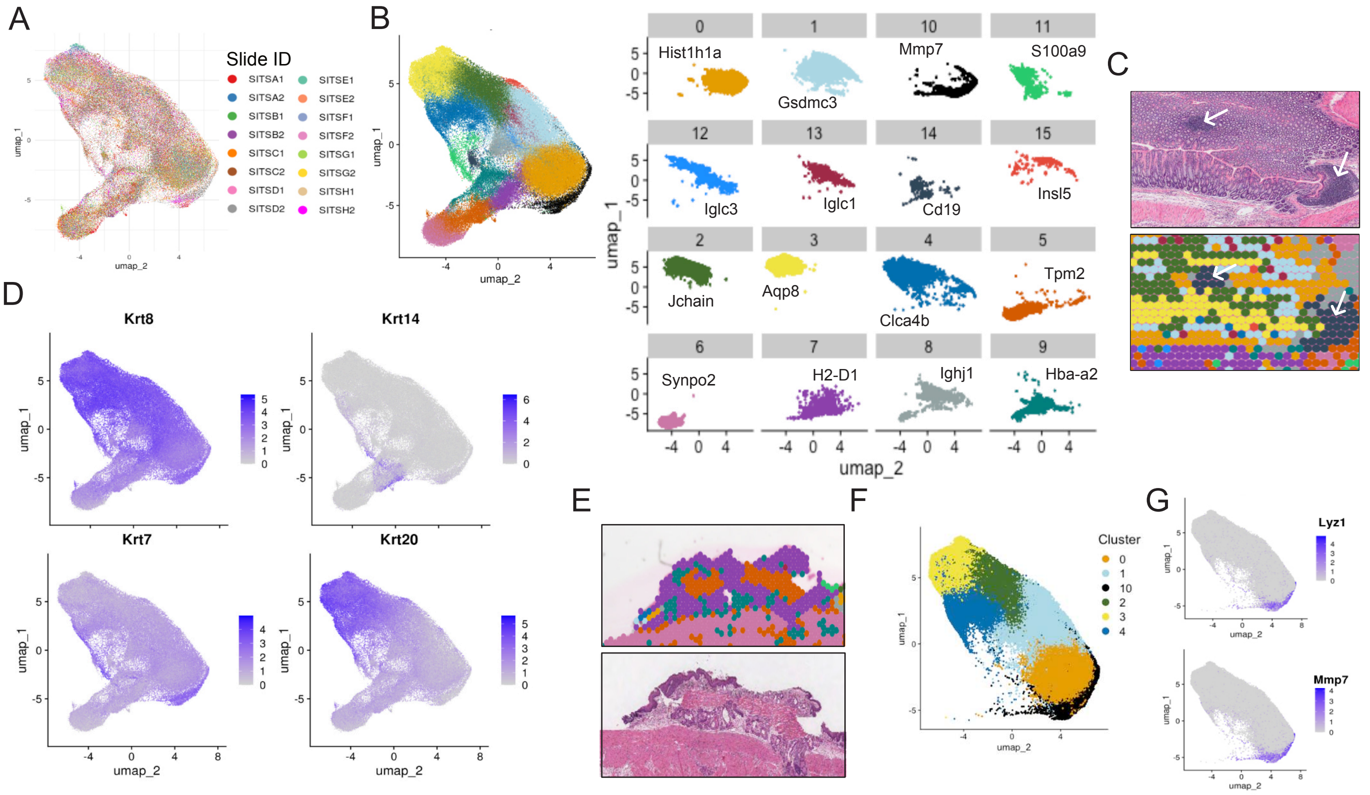

Supplementary Figure 3: Spatial transcriptomics detects cellular neighbourhoods in colitis and associated neoplasia 3. A) UMAP of barcodes coloured by slide, showing overlap after Harmony integration. B) Clusters represented in UMAP space with and without overlay, with top markers indicated (top 2 genes with highest log2 fold change and biggest differences in percentage barcode expression to other clusters). C) Clusters on spatial coordinates (top) and representative H&E image (bottom), arrows pointing to isolated lymphoid follicles marked by cluster 14. D) UMAPs showing distribution of expression of Krt8 (general epithelial marker), Krt14 (squamous epithelium), Krt7 (epithelial carcinogenesis) and Krt20 (healthy intestinal epithelium). E) Clusters on spatial coordinates (top) and representative H&E image (bottom) in an area containing muscle and squamous epithelium. F) UMAP showing the 5 main epithelial clusters (0-4) and tumour cluster (10). G) UMAPs showing distribution of expression of Lyz1 and Mmp7, markers of the tumour cluster.

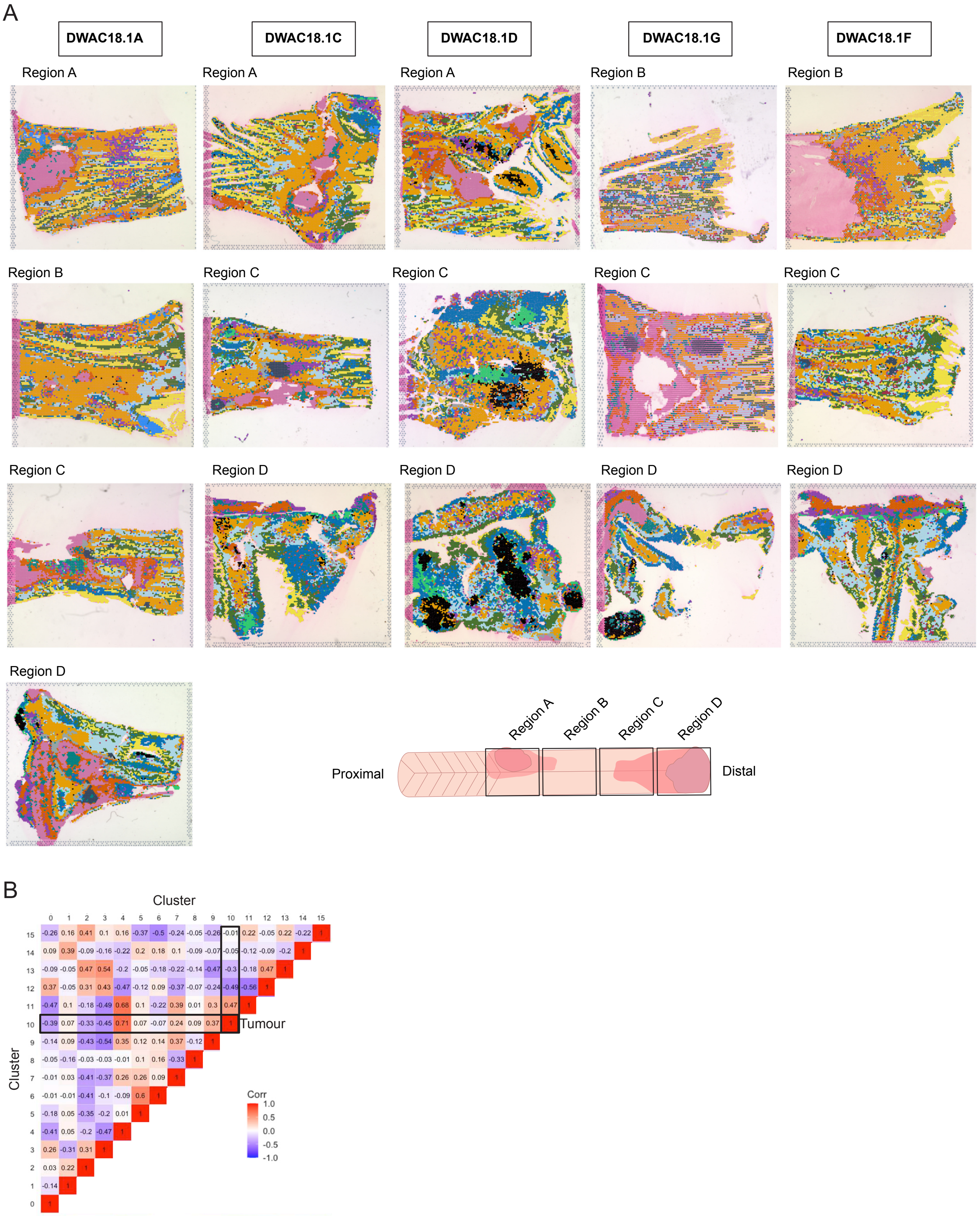

Supplementary Figure 4: Spatial representation and evolution of clusters. A) Clusters plotted onto spatial coordinates for each tissue sample, mapped onto the H&E-stained tissue. Mouse ID indicated at the top of each column; region indicated at the top of each picture. Drawing at the bottom shows the location of each region along colon. B) Matrix showing correlation in the coverage by each of the clusters in each tissue piece (Kendall rank correlation coefficient on plot, black boxes indicate correlation to the tumour cluster).

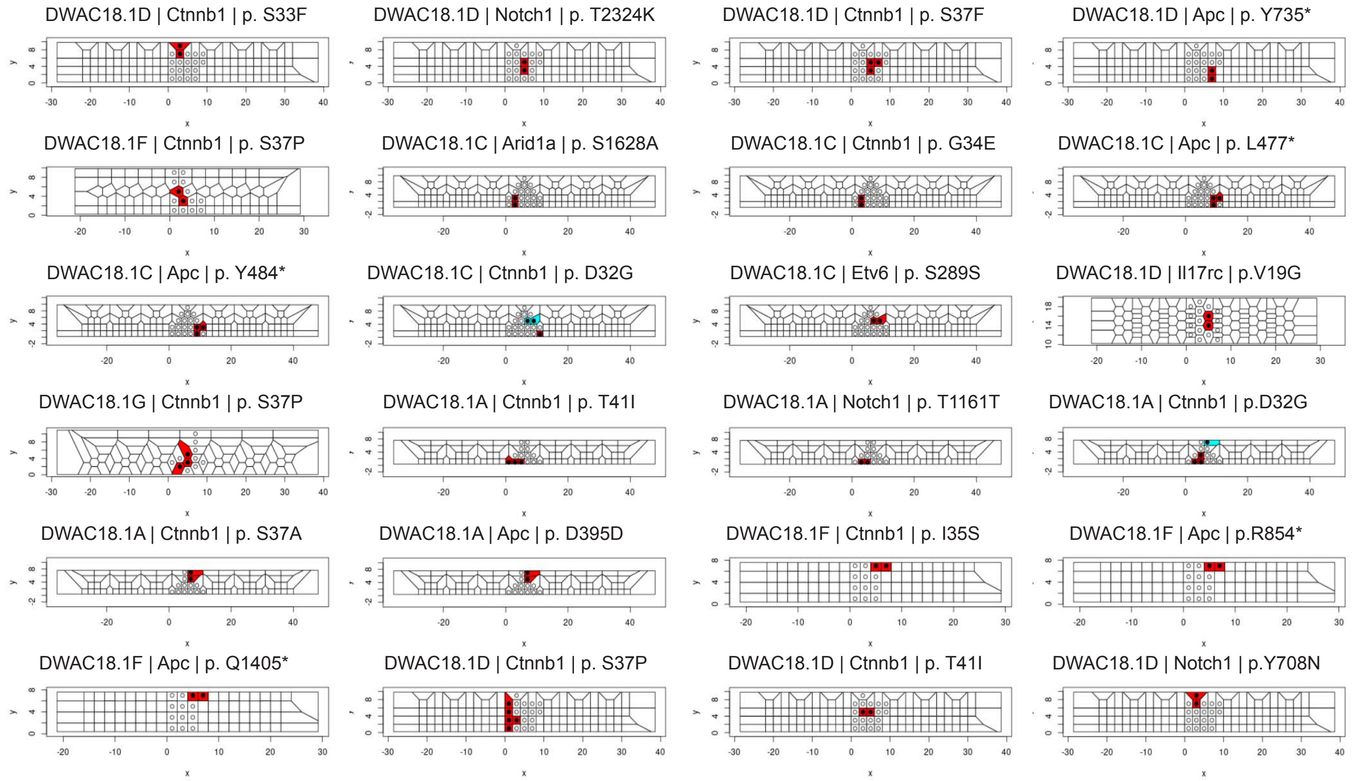

Supplementary Figure 5: Voronoi Tessellation of tissue sections for clone calling. Plots representing each piece of tissue where the same mutation was detected in at least 2 adjacent biopsies. Black dots represent biopsies taken for the relevant piece of tissue; colour represent clones (considered distinct if distance > 2mm) . Repeated patterns on either side show the Tessellation that was applied in order to detect adjacent cores at tissue borders.

A

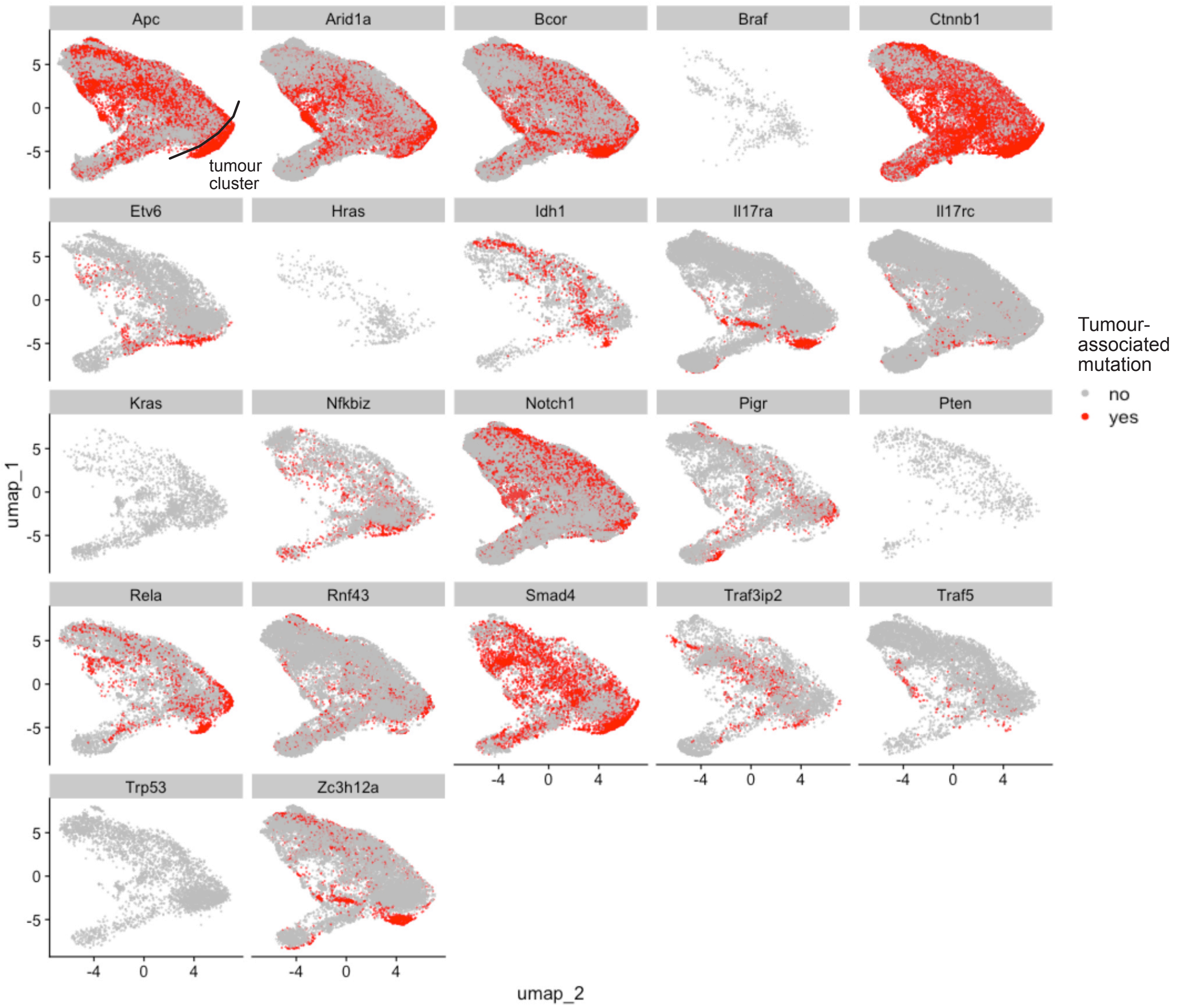

B

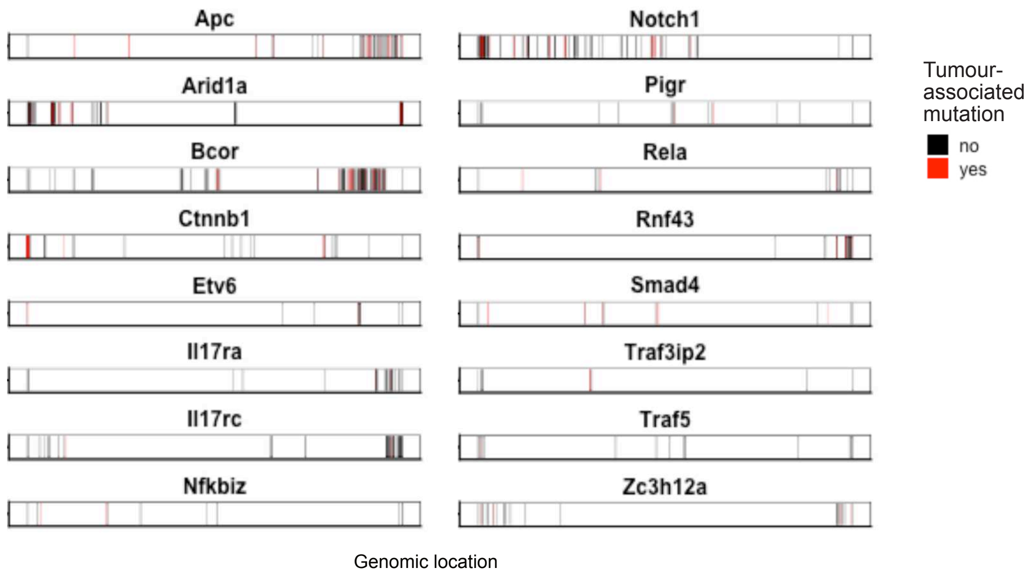

Supplementary Figure 6: Identifying tumour associated mutations. A) Distribution of tumour-associated and non-tumour-associated SNVs in each gene plotted on UMAP space (tumour cluster is circled in black in top left UMAP, size of dot indicates VAF). B) Tile plots showing distribution of tumour-associated and non-tumour-associated SNVs along each gene length (features genes with >10 mutations detected).

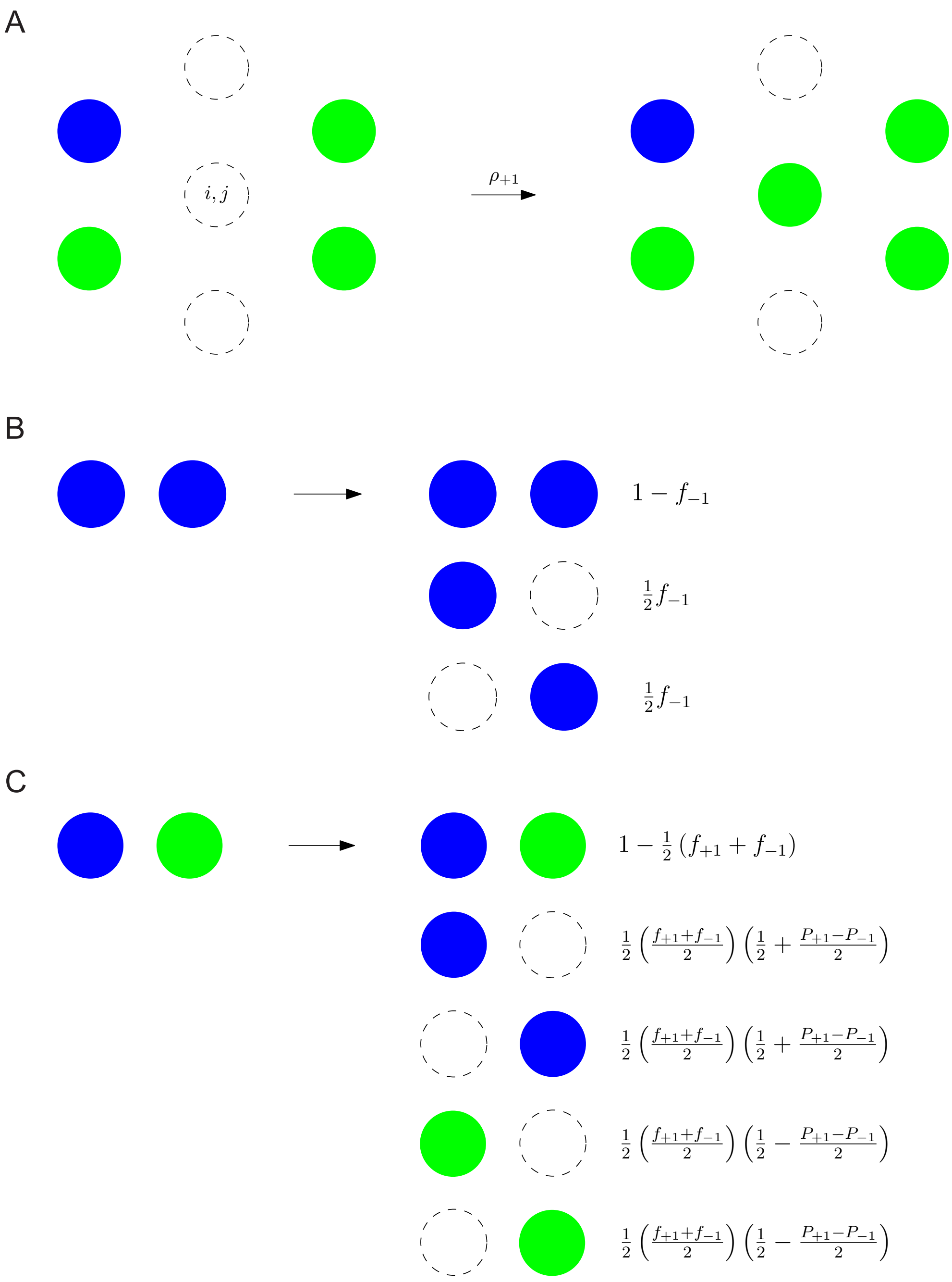

Supplementary Note 1: Possible outcomes of crypt dynamics simulations. A) Example of a fission event according to Rule 1(b) or 2(a) in the methods section. In this case a labelled site is the chosen neighbour and has probability to undergo fission and occupy the empty site. B) Example of a fusion event between two unlabelled sites according to Rule 2(b) (where the state is the same for both the chosen site and chosen neighbour). In the first case, no fusion occurs. In the second and third case, fusion occurs and leaves behind an empty site. C) Possible outcomes of fusion and monoclonal conversion according to Rule 2(b) where the chosen site and its chosen neighbour have different states. In the first case, no fusion occurs. In the remaining cases, fusion occurs with monoclonal conversion determining the final state according to the fixation probabilities as shown.
